# Development and validation of Yatt Suicide Attitude Scale (YSAS) in Malaysia

**DOI:** 10.1371/journal.pone.0209971

**Published:** 2019-02-27

**Authors:** Norhayati Ibrahim, Normah Che Din, Noh Amit, Shazli Ezzat Ghazali, Aisyah Mohd Safien

**Affiliations:** Health Psychology Programme, School of Healthcare Sciences, Faculty of Health Sciences, Universiti Kebangsaan Malaysia, Kuala Lumpur, Malaysia; University of Toronto, CANADA

## Abstract

**Introduction:**

Despite suicide rate becoming a growing trend in research locally and globally, there is no standard measuring instrument developed in Malaysia. The aim of this study is to establish the first ever Malay version of suicide screening tool that is suitable and fit with multiracial and complex culture of Malaysia.

**Methods:**

This study comprises of three phases, namely (1) items selection phase, (2) pilot study phase, and (3) scale validation phase. During the first phase, the items were selected from items pools which gathered from previous suicide ideation/ attitude scales. Then the pilot study was carried out to examine the items for Yatt Suicide Attitude Scale (YSAS). Lastly, the Yatt Suicide Attitude Scale (YSAS) validation study was conducted with 219 university students.

**Results:**

Initial version of YSAS comprised 16 items and three components. After factor analysis, the questionnaire was reduced into only two components (Suicide Ideation and Suicide Attempt) with 5 items each. Both of the components obtained high reliability value (.89 and.86 respectively) and the questionnaire accounted for 67.84% of the total variance.

**Conclusion:**

The analysis showed that YSAS has an acceptable reliability and validity for Malaysian population. Although these findings corroborate literature on development of suicide ideation assessment instrument for specific cultural context, there is a need to further examine its reliability with clinical population and general population of different cultural context in Malaysia.

## Introduction

Suicide has been gaining more spotlight from media and researchers as the alarming statistics for this problem shows growing trend every year. Statistic released by the World Health Organization revealed that over 800 000 people have died by suicide in 2012 and it was the second leading death in 15–29 years old [1]. Scowcrof [2] reported a suicide statistic in 2016 and stated that the rate was increasing among female victims globally as in UK (by 8.3%), England (by 14%), Scotland (by 7.8%), and Republic of Ireland (by 14.7%) between year 2013 and 2014. This trend is similar in Malaysia and it was reported that suicidal rate in Malaysia increased up till 60% over the past 45 years [3]. Malaysian Psychiatric Association estimated that seven people died daily by taking their own lives [4]. This horrifying numbers should be enough for everyone to step in and come out with protective and effective strategies in handling this sensitive issue.

According to Biodyne Model, suicide process involves three stages namely ideation, planning and autopilot [5] Although there is no consensus on the suicide terminologies over the past 50 years in the field of suicidology [6] but Biodyne Model is extensively used in research studies to describe development of suicide in oneself. The Cummings successfully includes what is need in treating the suicidal patient. The individual is said to be in suicidal ideation phase when he/she kept thinking about suicide continuously without designing a specific plan. They then find themselves engaging with dark thoughts and music and subsequently expressed it through arts. Planning stage is when that person begins to formulate suicidal plan. They began to detached themselves with their loved ones and may stop verbalizing the pain and suffering while seeming to be in more pain than ever. During this stage, the individual is in high emotional stress; to do or not to do suicide. It is a decision that most people do not discuss with loved ones and often wrestle by themselves alone. Person in autopilot stage, on the other hand, are consider imminent lethal as they usually attempt suicide within the next 48 hours. They appear calmer than usual as they already made up their mind and no longer wrestling with the decision [7]. The first and second stage may only linger in the victim’s thought, it is crucial for the authority and the researcher to develop the screening tool to recognize them. In a National Health and Morbidity Survey done by Ministry of Health Malaysia [8], it was found that 6.3% of the respondents having suicide ideation by themselves. A more shocking number was found by a group of researchers in study among adolescents attending government secondary schools in Malaysia. Four hundred and ninety three out of 1769 students responded that they were having suicidal ideation as they answered “several days”, “more than half of the day” or “nearly every day” to the questions [9].

These contradictory figures could be attributed to the fact that there was no standardized tool in screening this particular subject in Malaysia. A systematic reviewed article for suicide attempts in Malaysia from year 1969 to 2011 by Aishvarya and friends in 2014 [10] revealed that 6 from 38 studies used different established scales to measure suicidal behavior such as Suicide Intent Scale, Hopelessness Scale, Reasons for Living Inventory, but none of those were validated for use in Malaysian context. Meanwhile, Chan, Maniam and Shamsul [11] used Scale for Suicide Ideation (SSI) in their study.

Apart from the diverse scales used for this one particular subject, there is also an issue regarding the respondents involved. It was found that 63% of the respondents from 38 studies were those who admitted to the hospital after their suicide attempt and only 3% of them from hospital visits [10]. Similarly, the study by Chan et al [11] with inpatients respondents in hospital showed that one third of the subjects presented after a current suicide attempt. In other words, their target populations were only those people who already tried to kill themselves and prevented by the authority. 76% of the studies also only looked for sociodemographic data, psychiatric illness, and method and reasons for suicide attempts [10]. Looking from these perspectives, it is inadequate to make general view on Malaysian population with reference to suicide problem. Previous studies which only involved identified survivor of suicide made it inappropriate in establishing data on suicidal screening.

Malaysian government has taken a step forward in handling this problem by allocating a huge amount of about RM900 (USD 225) million from 2006 to 2010 in Ninth Malaysia Plan. It was to improve services for mental health problems including suicide and self-harm in Malaysia [12]. However, according to Armitage et. al [13], due to the bias of research studies on certain types of self-harm among specific ethnic groups, this huge budgets produced limited impact as the research literature into suicide and self-harm in Malaysia has been fragmented. Furthermore, based on the knowledge of the researcher, there has not been well-established suicidal behavior screening tools in Malaysian. Most of the previous suicide studies, as described before, usually use the western version of suicide assessment tools which might be ill-fitted when applied locally. This was what happened in a cross-cultural study of Attitude toward Suicide Questionnaire. Researchers found that the data taken from Malaysian respondents produced unequal amount of factors as compared with the original study in Western population [14]. Similar findings also obtained in earlier cross-cultural study on psychological instruments in Malaysia such as in Ng, Trusty & Crawford, [15], Ramli, Mohd Ariff, & Zaid [16] and Oei, Sukanlaya, Goh & Firdaus, [17]. Cultural bias has been discussed extensively since 1900s, when Binet’s first intelligence scale was published and Stern introduced procedures for testing intelligence [18, 19]. Williams (1970) and Helms (1992) proposed that two different groups of participants (black and white respondents) have qualitatively different cognitive structures, which must be measured using different methods (in Reynolds) [20]. Thus, the different between educational system and standard received by the Westerners compared to Asian, especially Malaysian, may contribute to different feedback from the questionnaire. Brown, Reynolds, and Whitaker [21] however believe that cultural bias due to racial or ethnic differences in test scores reflect “no real differences in ability, but rather problems in the construction, design, administration, or interpretation of tests” (p. 209). Reynolds [22] suggested three ways that a test or test item might be biased, from the content or topic of a question to the existence of one correct answer, as well as limited and specific vocabulary.

In Malaysia, Bahasa Melayu is the main lingua franca covering the urban and rural areas and is also the official language of the country [23]. Thus, it is crucial for the psychological testing to be in its spoken and written language. Although it is disputable as this issue can be solve through translation process, bear in mind that there were multiple translation procedures can be used for this particular purpose namely simple direct translation, modified direct translation, translation/back-translation, parallel blind technique, random probe and “ultimate” test [24]. Different kinds of translation may result into different sequence and types of words used in the sentence which eventually lead to dissimilar and unalike interpretation. Behling & Law [24] also stated that it is almost impossible to find translator who are fluent in both languages, expert in both cultures, and extremely knowledgeable in both contents measured.

The development of Malay version of suicidal behavior screening tool will thus help to contribute to the knowledge of suicidal behavior in Malaysian context. Ministry of Health Malaysia stated in their report that “National Suicide Registry Malaysia is studying the least common form of suicidal behavior: the completed suicides. Malaysia needs more data on non-fatal self-harm and suicidal ideations [8]. Due to suicide being a taboo topic in Malaysia [25], the development of suicide screening tool can help to identify and prevent suicide.

## Methods

This cross-sectional study was carried out on 219 students from Universiti Kebangsaan Malaysia from January to February 2018. University’s students were chosen for this study since young adults are those who mostly captivated in suicide problem. They were also a convenient sample for the researchers and lastly, this is validation study of a newly developed instrument. That is why it was done only on one section of the public, not in general population. This study was approved by the Universiti Kebangsaan Malaysia Research Ethics Committee (approval number NN-2018-060). The procedure was divided into two phases.

**Fig 1:**
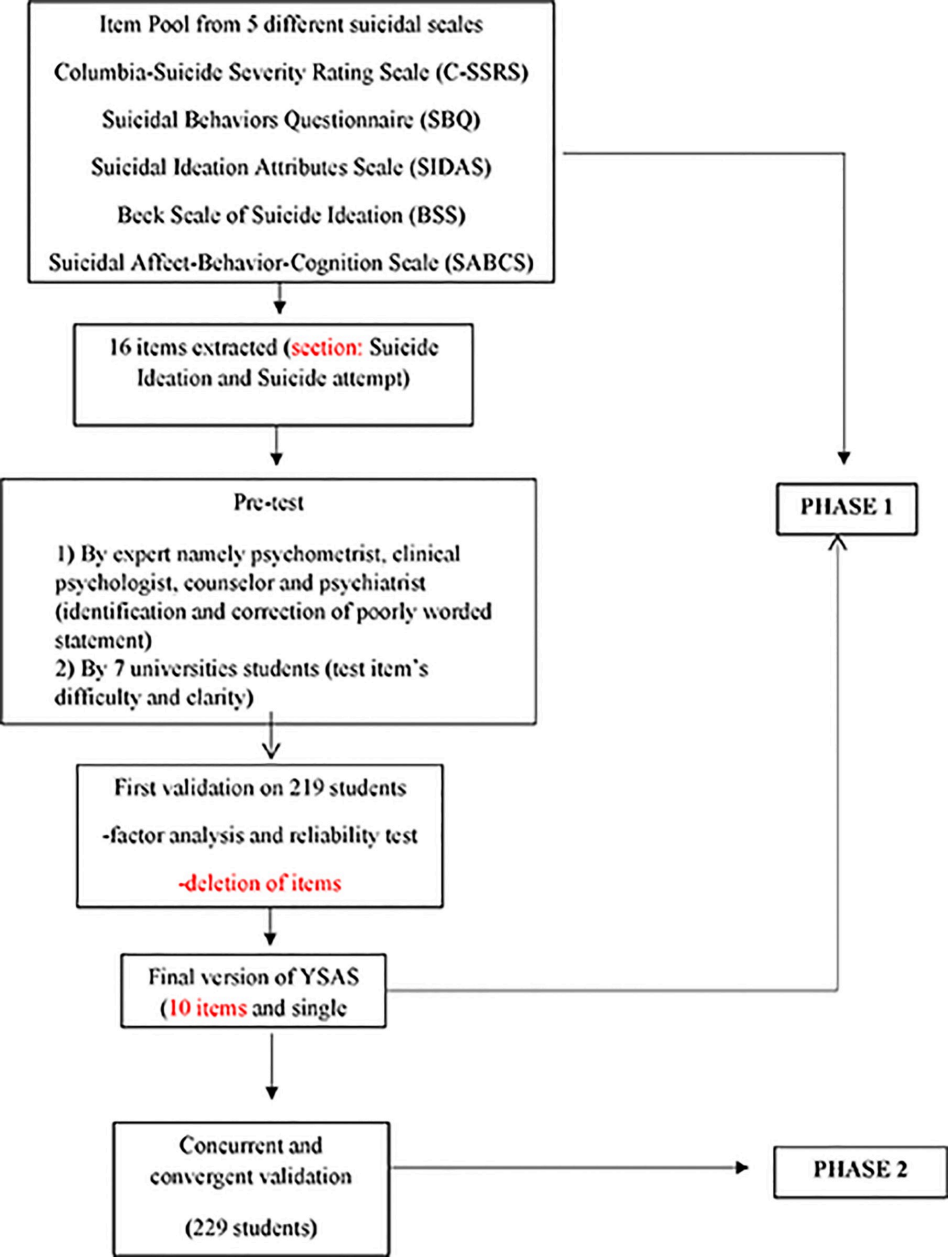
Flow chart for YSAS development.

Phase 1 of the YSAS development, as showed in Fig 1, was based on pool item derived from already developed suicide behavior and attitude scale. A total of 5 questionnaires were reviewed namely Columbia-Suicide Severity Rating Scale (C-SSRS) [26], Suicidal Behaviors Questionnaire (SBQ) [27], Suicidal Ideation Attributes Scale (SIDAS), [28], Beck Scale of Suicide Ideation (BSS) [29], and Suicidal Affect-Behavior-Cognition Scale (SABCS) [30].

The 16 items were considered suitable for the item pool and separated into two parts, suicide ideation and suicide attempt sections. Before YSAS were conducted on university students, pre-test of the item, the nature and consistency of the tool was carried out. The pre-test was conducted by having four experts in psychometrics and clinical psychologies, counselors and psychiatry to read and review the items in YSAS. In this stage unclear items or those that are poorly worded were identified and corrected. Furthermore, seven clinical students were asked about the items’ difficulty and clarity of each item.

YSAS was conducted on the first group of respondents involving 219 university’s students. Comrey and Lee [31] regarded 200 participants to be a fair sample size, while Guadagnoli and Velicer [32] believed that if several high loading (> 0.80) variables are present, then a big sample size is not required. Data collected then were analyzed using factor analysis and reliability test. Accordingly, inappropriate and low statistically items were deleted.

Lastly, in phase 2, the final suicide attitude scale which only left with 10 items and set into single division was tested for the second round on another 229 university students to reconfirm the results. It is also cross validated with Suicide Ideation Scale and Kessler’s K10 Psychological Distress Scale to get convergent and concurrent validity.

### Instruments

Two instruments namely Suicide Ideation Scale (SIS) and Kessler’s K10 Psychological Distress Scale (K10) was used in the validation of the newly developed questionnaire. SIS was chosen to determine convergent validity of YSAS. According to Campbell & Fiske, [33], convergent validity coefficients are the correlations between measures of the same trait that are obtained with different measurement methods. For this reason, as YSAS is developed to screen suicide ideation and attempt of an individual, SIS is a good choice to be cross validated. Researchers also ran concurrent validity using K10 scale since psychological stress is a well-known factor related to suicide [34–37]. Cronbach & Meehl [38] stated that concurrent validity is done to see relation between a test with some contemporary criterion.

#### Suicide Ideation Scale (SIS)

The Suicide Ideation Scale was developed by Rudd [39]. This scale consisted of 10 items to measure the level of suicide ideation or intention among adolescents and adults. Each item had five Likert options of 1 (never) to 5 (many times). SIS has high internal consistency (Cronbach alpha = .86) as well as adequate item-total correlations ranging from.45 to.74 [39].

#### Kessler’s K10 psychological distress scale

The K10 scale is a short instrument to measure psychological distress levels such as depression and anxiety, which was designed by Kessler et al. [40]. Although K10 was originally developed to identify non-specific psychological distress in the general population [41] it was also has been used extensively in specialist public mental health services across Australian states and territories [42]. It comprises of ten questions pertaining to the respondent’s emotional state within the last month and uses a cut off score of 20 to determine whether the respondent is likely to be distressed. The reliability value of the scale was 0.93 in a sample of caregivers of cancer patients in Guam, USA [43] and 0.87 in Malaysia among caregiver with schizophrenia patients [44], and 0.91 among first semester university students [45].

### Data analysis

The raw data were keyed into the SPSS version 21. Demographic data were obtained from descriptive analysis. All of the items underwent exploratory factor analysis to confirm number of factors while reliability of the questionnaire was acquired from the Cronbach alpha value. To measure the content validity of the questionnaire, Pearson Correlation analysis was used.

## Results

The scale was distributed to 219 students of Universiti Kebangsaan Malaysia. As indicated in Table 1, more than 80% of them were female (83.1%). From 142 respondents, 64.84% of the students were between 18–19 years old and the rest between 20–25 years old. Malay contributes for the majority of the respondents, followed by Chinese, Indian and others with their percentages were 63%, 27.9%, 7.3% and 1.8% respectively. The results showed that most of the respondents came from family with RM1001-RM3000 (USD241—USD723) household incomes (45.2%) and the least of them were from no income family (0.9%). Majority of the respondents were from intermediate size of family with 4–6 siblings (46.12%) and were among the earliest children of their parents (71.23%).

**Table 1:** Demography data.

Characteristics (n = 219)	Frequency	Percentage (%)
Gender		
Male	37	16.9
Female	182	83.1
Age		
18–19 years old	142	64.84
20–21 years old	68	31.05
22–25 years old	9	4.11
Race		
Malay	138	63.0
Chinese	61	27.9
Indian	16	7.3
Others	4	1.8
Family income		
<RM1000 (<USD241)	22	10.0
RM1001-3000 (USD241-723)	99	45.2
RM3001-5000 (USD723-1204)	41	18.7
>RM5000 (>USD1204)	55	25.1
No income	2	0.9
Numbers of Sibling		
1–3	94	42.92
4–6	101	46.12
7–9	19	8.68
10–12	5	2.28
Birth Order		
1–3	156	71.23
4–6	56	25.57
7–9	5	2.28
10–12	2	0.91

The two sub constructs of the instrument, initially with 8 items each (in phase 1), were analysed separately. Researchers ran exploratory factor analysis to establish underlying dimensions between measured variables and constructs. Principal component and varimax rotation analyses were used on the data of 219 respondents. Kaiser–Meyer-Olkin Measure of Sampling Adequacy value was.83 which is above the recommended value of.60 and closes to 1.0 indicates that the variables share common factors and can be factorized. Bartlett’s test of Sphericity value for the scale is.000 (below.05) and denotes that the responses collected is appropriate to the problems addressed in the study.

Factor loading for each item of the scale were showed in Table 2. Items in Suicide Ideation section fall perfectly into one component with all of the items obtained loading value more than.40 and the total variance explained by the scale is 49.27%. On the other hand, items in Suicide Attempt section divided into two components where item 1, 2, 3, 5, 7 and 8 were in the first component and item 4 and 6 in the second component. Cumulative variance for Suicide Attempt was up to 67.23%.

**Table 2:** Exploratory factorial analysis of YSAS (varimax rotation with kaiser normalisation).

Factors	Factor loading	Eigen value	Explained Variance %
16 items YSAS			
**Suicide Ideation section**			
**Component 1**		3.94	46.27
1. Saya tidak ada keinginan untuk… *I have no will to…*	.65		
2. Saya pernah merancang cara tertentu untuk… *I have come up with certain ways to…*	.48		
3. Saya merasakan tidak ada sebab untuk … *I have felt like there is no reason to…*	.77		
4. Terlintas dalam fikiran saya untuk menamatkan hidup saya apabila… *It has crossed my mind to end my life when…*	.71		
5. Saya pernah terfikir untuk… *I have once thought to…*	.83		
6. Saya merasakan tidak ada jalan penyelesaian terhadap masalah saya melainkan… *I feel that there is no solution to my problem but to…*	.64		
7. Saya pernah terfikir untuk berbuat sesuatu agar… *I once thought of doing something so…*	.73		
8. Terlintas dalam fikiran saya untuk menamatkan hidup saya namun … *It has crossed my mind to end my life but…*	.77		
**Suicide Attempt Section**			
**Component 1**		4.03	50.39
1. Saya pernah mencederakan diri saya sendiri dengan… *I have harmed myself for the purpose…*	.78		
2. Saya pernah menggunakan kaedah tertentu untuk… *I have tried certain ways to…*	.89		
3. Saya pernah melakukan percubaan untuk … *I have tried to…*	.91		
5. Saya pernah mencuba untuk menamatkan hidup saya tetapi… *I have tried to end my life but…*	.76		
7. Saya risau yang saya akan melakukan cubaan untuk… *I am worried that I would try to…*	.50		
8. Saya sudah mencuba menamatkan hidup saya tetapi… *I have attempted to end my life but…*	.79		
**Component 2**		1.35	67.23
4. Saya tidak berupaya menghalang diri daripada … *I have been unable to prevent myself from…*	.75		
6. Saya merasakan hidup ini tidak ada makna sehingga… *I feel as if this life has no meaning that…*	.80		
10 items YSAS			
**Component 1**		4.891	48.91
Saya pernah mencederakan diri sendiri dengan tujuan untuk… *I have hurt myself for the purpose of…*	.67		
Saya pernah menggunakan kaedah tertentu untuk… *I have tried certain methods to…*	.87		
Saya pernah melakukan percubaan untuk menamatkan hidup saya tetapi … *I have tried to end my life but…*	.93		
Saya pernah mencuba untuk menamatkan hidup ini tetapi … *I have tried to end my life but…*	.84		
Saya pernah mencuba menamatkan hidup saya tetapi sebenarnya… *I have attempted to end my life but…*	.79		
**Component 2**		1.893	67.84
Saya tidak ada keinginan untuk… *I have no will to…*	.70		
Saya merasakan tidak ada sebab untuk… *I feel like there is no reason for me to…*	.80		
Terlintas dalam fikiran saya untuk menamatkan hidup ini apabila … *It has crossed my mind to end my life when…*	.77		
Saya pernah terfikir untuk… *I have once thought to…*	.78		
Terlintas dalam fikiran saya untuk menamatkan hidup saya namun… *It has crossed my mind to end my life but…*	.71		

As the cumulative variance for Suicide Ideation did not reach even half (only 49.27%), researchers reviewed the items and concludes that item 2, 6 and 7 did not describe Suicide Ideation but rather the planning process. These items were deleted which eventually increased its cumulative variance up to 60.42%. Meanwhile, for Suicide Attempt section, item 4 and 6 were checked and considered inappropriate to explain the variable. They were removed together with item 7 as its factor loading value was the lowest. Suicide Attempt section then left with single component and its cumulative variance then increased up to 72.28%. All of these cumulative variances showed in Table 3 together with alpha Cronbach value. Deletion of the items results into the final version of YSAS with 10 items only. Its factor analysis brings out single component for each of the construct as showed in Table 2.

**Table 3:** Alpha Cronbach and cumulative percentage summary.

Sub construct	Numbers of item		Cronbach alpha		% Cumulative percentage	
	Before	After	Before	After	Before	After
Suicide ideation	8	5	.83	.83	49.27	60.42
Suicide attempt	8	5	.79	.89	67.23	72.28
Suicide Attitude (SI + SB)	16	10	.86	.84	60.66	67.84

After deleting item 2, 6 and 7 from suicide ideation subconstruct, its Cronbach alpha increased from.79 to.89 as indicated in Table 3. When suicide attempt subscale underwent the same procedure by removing item 4, 6 and 7, its reliability coefficient remained the same (.83 to.83) but its cumulative percentage was raised which is more favorable to the study. For cumulative percentage, 10-item YSAS described 67.84% of the required themes after deleting items mentioned as compared to only 60.66% before by 16-item YSAS. Cronbach alpha value also did not differ too much from the previous value. Although the reliability coefficient was reduced (.84) compared to 16-items instrument (.86), cumulative variance value of the questionnaire is more crucial.

To further validate the scale, researcher conducted Pearson correlation analysis between the scale with another questionnaire that were already developed involving 229 university’s students in phase 2. Table 4 showed that both Suicide Ideation and Suicide Attempt subconstructs showed significant and positive correlation with Suicide Ideation Scale (r = .63, p = .00; r = .43, p = .00) and Psychological Distress variable (r = .41, p = .00; r = .18, p = .01). Lastly, Yatt Suicide Attitude Scale established an accepted convergent and concurrent validity as it obtained significant correlation with Suicide Ideation Scale (r = .64, p = .00) and Psychological Distress (r = .38, p = .00).

**Table 4:** Concurrent and convergent validity of YSAS.

	Psychological Distress (concurrent)		Suicide Ideation Scale (convergent)	
	P value	Correlation coefficient	P value	Correlation coefficient
Suicide Ideation	.000	.41**	.000	.63**
Suicide Attempt	.000	.18**	.000	.43**
Yatt Suicide Attitude Scale (YSAS) (SI +SB)	.000	.64**	.000	.38**

**. p <.01 two-tailed

Due to the fact that ratio of male to female respondents is not balanced, researchers decided to ran analysis only on the female participants. Findings written in Table 5 showed that similar results were obtained before and after deletion 6 of the items mentioned above. Two components from suicide attempt subscontruct reduced into one while all items from suicide ideation section also grouped into one component from the start. Cumulative percentage was also increased for both subscontruct after removal of the items.

**Table 5:** Analysis summary for female respondents only.

Sub construct	Numbers of item		Cronbach alpha		% Cumulative percentage	
	Before	After	Before	After	Before	After
Suicide ideation	8	5	.83	.82	50.15	60.54
Suicide attempt	8	5	.78	.88	66.68	71.81
Suicide Attitude (SI + SB)	16	10	.87	.85	61.87	68.28

## Discussion

The main purpose of this study was to developed a suicide screening tool for Malaysian population. The scale was designed to measure the two stages of suicidal process on separate subscales (suicidal ideation and suicidal attempt), with the intention to summing the subscales scores to create a total score describing participant’s overall suicide attitude. Incorporation of both these subscales were theoretically motivated so that they can be reflected in the scale design. The final YSAS questionnaire was composed of two components of Suicide Attitude, consisted 10 items with high Cronbach alpha value which above.80 for both of them.

The results of the items intended to explain the two components of suicidal process loaded into three factors rather than two were unexpected. This is because all of the items were extracted from already established scales available. But, according to Enos [46] and Finney [47], it is a typical outcome for negative and positive items of self-report measures to load on separate factors. Factor analysis for Suicide Attempt section in the first phase resulted in two components with the seventh item of suicide attempt subscale had the lowest factor loading value. The item was then deleted. As only item 4 and 6 of suicide attempt subscale contributes to the second component of the scale, researchers then revised and re-evaluated both of the items. Upon looking to the item, researchers believed that the items did not explain the construct directly and clearly, so they were deleted.

For suicide ideation subscale, all 8 items fall perfectly in one factor. However, cumulative variance percentage was not even half. Researcher decided to remove some items after re-evaluation even though all of them were above range 0.40. Item 2, 6 and 7 were selected to be removed and resulted into a higher percentage of cumulative variance from 49.27% to 60.42%, as well as better alpha Cronbach value (.79 to.89).

In terms of reliability analysis, both of subscales and YSAS itself showed high Cronbach alpha after deletion of the items. As a conclusion, deleting of the items depended on Cronbach alpha value of the subscale as well as cumulative variance percentage. But when there were frictions in between them, the later was prioritized as the difference of before and after reliability value was minute and still in the same range of strength.

After removal 6 of the items, final version of YSAS questionnaire was obtained. Researcher then further analyzed the instrument by doing criterion-related validation by looking on convergent and concurrent validity. Campbell and Fiske [33] concluded that the most common approach to establishing convergent and discriminant validity is to demonstrate that multiple measures of a construct are related, and more related to each other than to measures other constructs, even when the two measurement methods are similar. Hence, convergent validity was done to find the relationship between YSAS with another established questionnaire on suicide theme namely Suicide Ideation Scale by Rudd. Since Rudd also measures the whole concept of suicide and not limited to only ideation or attempt, it correlates higher with YSAS compared to both of the subscales individually. This is in line with Widaman [48] who believed that although there were various means to measure the same trait, it should correlate higher as it evaluates the same thing. Similarly, Nunnally [49] proposed that convergent validity is directly proportional to correlation. Highly correlated measures mean that it is valid highly in convergences, whereas measures that are correlated close to zero suggest weak or no convergence.

Development of instruments targeted specifically for Malaysian is important as most of readily available questionnaires were from the West and from Western context. This is because, firstly, Western based suicide instruments were developed and conducted on races in the West like native American, American Hispanic, American African, to name a few, as were in Heisel & Flett, [50], Lamis & Lester [51], Beck, Kovacs & Weissman [52] and Rudd [39]. This is already distinct to the inherent ethnics in Malaysia. Different culture exhibits difference traditional values, norms and practice [53, 54]. Inaccurate translation of English format questionnaire into another language may also contribute to the insufficient understanding of the items [55, 56] and does not reflect what the item should measure which subsequently will provide poor data quality to the researchers [57].

## Strength and limitation

This questionnaire can be considered as the first instrument in measuring suicide behavior among Malaysian as it has been developed within Malaysian context as well as tested on Malaysian population. Heterogeneity of participants is half achieved as the respondents involved were only from the youth and young adult who were above 18 years old. Also, majority of the respondents in this validation study were female and this limit the generalization of the tool. This questionnaire is applicable for multiracial nation of Malaysia as all major ethnicity were involved in the study. Another limitation for this study was that, respondents contributed to data collection who were university students whose cognitive function and language literacy were good. The level of education also influences subject’s openness towards this sensitive issue among the community. Furthermore, as the respondents were only university students, they were usually supported financially by their parents or sponsors who made them unaware of monetary burden. Financial crisis has always been one of the major reasons for ending one’s life [58, 59]. Thus, a more generalized future study incorporated different types of populations and setting is required to further explore and improve validity and reliability of this suicide screening tool so that it can be used in Malaysia.

## Conclusion

The 10-item Yatt Suicide Attitude Scale (YSAS) was proven to be an acceptable and trustworthy instrument used in screening suicide behavior in Malaysia. The scale underwent several analyses to confirm its reliability and validity, and it is thereby suitable to be used in future suicide study especially in the Malaysian context. This questionnaire may assist prospective fellow researcher to have better overview of suicide dilemma among Malaysian. It is also expected to be a supportive device for the government to further improve methods in handling this serious matter, not only by screening the victims out but also in treating them.

## Supporting information

S1 File Instrument Attachment for Plos One. (DOCX)

S2 File Yatt Suicide Attitude Scale (YSAS). (DOCX)
