# Supplementary material for: Development and validation of Yatt Suicide Attitude Scale (YSAS) in Malaysia
Source: PLoS One. 2019 Feb 27;14(2):e0209971. doi: 10.1371/journal.pone.0209971 (PMC6392240; doi:10.1371/journal.pone.0209971)
Supplement: S1 File — (DOCX) [file pone.0209971.s001.docx]

**16-item version of YSAS**

**BAHAGIAN A: Ideasi Bunuh Diri**

***SECTION A: Suicide ideation***

Pernyataan di bawah menerangkan tentang pemikiran atau percubaan untuk membunuh diri. Sila bulatkan pernyataan yang menggambarkan situasi anda.

*The statement below explains thoughts or attempts to commit suicide. Please tick a statement that describes your situation.*

| No | Item | Tidak pernah  *Never* | Kadang kadang  *Sometimes* | Selalu  *Always* | Kerap  *Frequent* | Sangat kerap  *Very often* |
| --- | --- | --- | --- | --- | --- | --- |
| 1 | Saya tidak ada keinginan untuk meneruskan kehidupan saya  *I have no will to continue my life.* |  |  |  |  |  |
| 2 | Saya pernah merancang cara tertentu untuk menamatkan hidup saya  *I have come up with certain ways to end my life.* |  |  |  |  |  |
| 3 | Saya merasakan tidak ada sebab untuk saya terus hidup.  *I have felt like there is no reason for me to live.* |  |  |  |  |  |
| 4 | Terlintas dalam fikiran saya untuk menamatkan hidup saya apabila saya berhadapan dengan masalah yang besar.  *It has crossed my mind to end my life when I am faced with a big problem.* |  |  |  |  |  |
| 5 | Saya pernah terfikir untuk menamatkan hidup saya.  *I have once thought to end my life.* |  |  |  |  |  |
| 6 | Saya merasakan tidak ada jalan penyelesaian terhadap masalah saya melainkan menamatkan hidup saya.  *I feel that there is no solution to my problem but to end my life.* |  |  |  |  |  |
| 7 | Saya pernah terfikir untuk berbuat sesuatu agar saya tidak lagi hidup pada keesokan harinya.  *I once thought of doing something so that I would no longer live the next day.* |  |  |  |  |  |
| 8 | Terlintas dalam fikiran saya untuk menamatkan hidup saya namun saya takut untuk melakukannya.  *It has crossed my mind to end my life but I am afraid to act upon it.* |  |  |  |  |  |

**BAHAGIAN B: Percubaan Bunuh Diri**

***SECTION B: Suicide Attempt***

Pernyataan di bawah menerangkan tentang pemikiran atau percubaan untuk membunuh diri. Sila bulatkan pernyataan yang menggambarkan situasi anda.

*The statement below explains thoughts or attempts to commit suicide. Please tick a statement that describes your situation.*

| No | Item | Tidak pernah  *Never* | Kadang kadang  *Sometimes* | Selalu  *Always* | Kerap  *Frequent* | Sangat kerap  *Very Often* |
| --- | --- | --- | --- | --- | --- | --- |
| 1 | Saya pernah mencederakan diri saya sendiri dengan tujuan untuk menamatkan hidup saya.  *I have harmed myself for the purpose of ending my life.* |  |  |  |  |  |
| 2 | Saya pernah menggunakan kaedah tertentu untuk menamatkan hidup saya.  *I have tried certain ways to end my life.* |  |  |  |  |  |
| 3 | Saya pernah melakukan percubaan untuk menamatkan hidup saya.  *I have tried to end my life.* |  |  |  |  |  |
| 4 | Saya tidak berupaya menghalang diri daripada mencederakan diri sendiri apabila dalam keadaan tertekan.  *I have been unable to prevent myself from self-injuring when I am under stress.* |  |  |  |  |  |
| 5 | Saya pernah mencuba untuk menamatkan hidup saya tetapi tidak berhasil.  *I have tried to end my life but it did not work.* |  |  |  |  |  |
| 6 | Saya merasakan hidup ini tidak ada makna sehingga saya pernah melakukan cubaan untuk menamatkan hidup saya.  *I feel as if this life has no meaning that I have tried to end it.* |  |  |  |  |  |
| 7 | Saya risau yang saya akan melakukan cubaan untuk menamatkan hidup saya lagi.  *I am worried that I would try to end my life again.* |  |  |  |  |  |
| 8 | Saya sudah mencuba menamatkan hidup saya tetapi saya tidak berkeinginan untuk mati.  *I have attempted to end my life but I did not want to die.* |  |  |  |  |  |

**10-item version of YSAS**

Pernyataan di bawah menerangkan tentang pemikiran atau percubaan untuk membunuh diri. Sila bulatkan pernyataan yang menggambarkan situasi anda.

*The statement below explains thoughts or attempts to commit suicide. Please tick a statement that describes your situation.*

| No | Item | Tidak pernah  Never | Kadang kadang  Sometime | Selalu  Always | Kerap  Frequent | Sangat kerap  Very Often |
| --- | --- | --- | --- | --- | --- | --- |
| 1 | Saya tidak ada keinginan untuk meneruskan kehidupan ini.  *I have no will to continue my life.* |  |  |  |  |  |
| 2 | Saya merasakan tidak ada sebab untuk saya terus hidup.  *I feel like there is no reason for me to live.* |  |  |  |  |  |
| 3 | Terlintas dalam fikiran saya untuk menamatkan hidup ini apabila berhadapan dengan masalah yang besar.  *It has crossed my mind to end my life when I am faced with a big problem.* |  |  |  |  |  |
| 4 | Saya pernah terfikir untuk menamatkan hidup saya.  *I have once thought to end my life.* |  |  |  |  |  |
| 5 | Terlintas dalam fikiran saya untuk menamatkan hidup saya namun saya takut untuk melakukannya.  *It has crossed my mind to end my life but I am afraid to do so.* |  |  |  |  |  |
| 6 | Saya pernah mencederakan diri sendiri dengan tujuan untuk menamatkan hidup saya.  *I have hurt myself for the purpose of ending my life.* |  |  |  |  |  |
| 7 | Saya pernah menggunakan kaedah tertentu untuk menamatkan hidup saya.  *I have tried certain methods to end my life.* |  |  |  |  |  |
| **8** | Saya pernah melakukan percubaan untuk menamatkan hidup saya tetapi menghentikannya apabila teringat tentang sesuatu (orang tersayang, dosa dll)  *I have tried to end my life but ceased to do so when I am remindered by something (loved ones, sins etc.)* |  |  |  |  |  |
| 9 | Saya pernah mencuba untuk menamatkan hidup ini tetapi tidak berhasil.  *I have tried to end my life but it did not work.* |  |  |  |  |  |
| 10 | Saya pernah mencuba menamatkan hidup saya tetapi sebenarnya saya tidak berkeinginan untuk mati*.*  *I have attempted to end my life but I actually did not want to die.* |  |  |  |  |  |
