# Supplementary material for: Development and validation of Yatt Suicide Attitude Scale (YSAS) in Malaysia
Source: PLoS One. 2019 Feb 27;14(2):e0209971. doi: 10.1371/journal.pone.0209971 (PMC6392240; doi:10.1371/journal.pone.0209971)
Supplement: S2 File — (DOCX) [file pone.0209971.s002.docx]

Alat Ujian Yatt Suicide Attitude Scale (YSAS)

Pernyataan di bawah menerangkan tentang pemikiran atau percubaan untuk membunuh diri. Sila bulatkan pernyataan yang menggambarkan situasi anda.

| **No** | **Soalan** | **Tidak pernah** | **Kadang**  **kadang** | **Selalu** | **Kerap** | **Amat kerap** |
| --- | --- | --- | --- | --- | --- | --- |
| 1 | Saya tidak ada keinginan untuk meneruskan kehidupan ini. |  |  |  |  |  |
| 2 | Saya merasakan tidak ada sebab untuk saya terus hidup. |  |  |  |  |  |
| 3 | Terlintas dalam fikiran saya untuk menamatkan hidup ini apabila berhadapan dengan masalah yang besar. |  |  |  |  |  |
| 4 | Saya pernah terfikir untuk menamatkan hidup saya. |  |  |  |  |  |
| 5 | Terlintas dalam fikiran saya untuk menamatkan hidup saya namun saya takut untuk melakukannya. |  |  |  |  |  |
| 6 | Saya pernah mencederakan diri sendiri dengan tujuan untuk menamatkan hidup saya. |  |  |  |  |  |
| 7 | Saya pernah menggunakan kaedah tertentu untuk menamatkan hidup saya. |  |  |  |  |  |
| 8 | Saya pernah melakukan percubaan untuk menamatkan hidup saya tetapi menghentikannya apabila teringat tentang sesuatu (orang tersayang, dosa dll) |  |  |  |  |  |
| 9 | Saya pernah mencuba untuk menamatkan hidup ini tetapi tidak berhasil. |  |  |  |  |  |
| 10 | Saya pernah mencuba menamatkan hidup saya tetapi sebenarnya saya tidak berkeinginan untuk mati. |  |  |  |  |  |
